# Changes in neural processing and evaluation of negative facial expressions after administration of an open-label placebo

**DOI:** 10.1038/s41598-022-10567-4

**Published:** 2022-04-21

**Authors:** Anne Schienle, Isabella Unger, Daniela Schwab

**Affiliations:** Department of Clinical Psychology, University of Graz, BioTechMed, Universitätsplatz 2/DG, 8010 Graz, Austria

**Keywords:** Physiology, Psychology

## Abstract

A recent event-related potential (ERP) study found that an open-label placebo (OLP) reduced emotional distress during the viewing of unpleasant scenes and the amplitude of the late positive potential (LPP). The present ERP experiment aimed at a conceptual replication of this finding and investigated OLP effects during affective face processing. The participants (109 females) were presented with images depicting angry and neutral facial expressions after the administration of a saline nasal spray. The spray was either introduced as a placebo that could help reduce the emotional reactions to viewing angry faces (OLP group) or to improve the electrophysiological recordings (Control group). The OLP was associated with reduced LPP amplitudes (1000–6000 ms) to anger expressions across a frontal cluster. Additionally, the OLP reduced LPP amplitudes (400–1000 ms) to both anger and neutral faces across a centroparietal cluster. Compared to the Control group, the OLP group reported less arousal when confronted with angry faces, and rated the anger expressions as less intense. This study demonstrates that an OLP can alter both subjective and neural responses to anger cues. Future research should directly compare OLP treatment with other strategies for emotion regulation (e.g., cognitive reappraisal) to demonstrate the specificity of this approach.

## Introduction

Placebos can alleviate emotional distress^1–8^. Numerous studies with functional magnetic resonance imaging (fMRI) and electroencephalography (EEG) have shown that placebo pills prescribed as psychotropic drugs (e.g., antidepressants, anxiolytics) or herbal medicine are able to reduce depression symptoms^1^, social pain^2^, and feelings of fear^3–5^ and disgust^6–8^ by altering activity in specific neural networks.

The mentioned studies administered placebos with deceptive suggestions. This approach has ethical issues that can be circumvented by using open-label placebos (OLPs). The recipients of OLPs are explicitly advised that they receive a sham intervention^9^. Several randomized controlled trials have already indicated that OLPs can reduce emotional distress in healthy participants^10–12^.

The neurophysiological mechanisms underlying OLP effects on affective processing are widely unknown. There is only one OLP study^11^ during which the participants were randomly assigned to either a placebo group or a control group (CG). Both groups first inhaled a saline nasal spray and were then presented with unpleasant and neutral scenes. While the OLP group was told that the spray (with no active ingredients) would help reduce the negative emotional reactions to the distressing images, the control group was informed that the spray would improve the electrophysiological recordings. It was found that the OLP reduced self-reports of emotional distress and amplitudes of the centroparietal LPP (late positive potential: 1000–6000 ms after picture onset) to both unpleasant and neutral images. The interaction between condition (placebo vs. no placebo) and picture type (negative vs. neutral) was not statistically significant. Therefore, the authors concluded that the OLP exerted a general dampening effect on emotional reactivity.

The current preregistered randomized EEG study aimed at a conceptual replication of the experiment by Guevarra et al^11^. A similar design and rationale was used to identify the primary effect of interest: an OLP-related reduction of emotional distress and late positivity while viewing negative affective pictures. The participants viewed pictures (for 6000 ms each) showing persons with angry or neutral facial expressions. Ratings for valence, arousal, and perceived anger intensity were recorded. Before the picture viewing, a presentation either summarized findings from neurobiological placebo research (OLP group) or affective neuroscience (CG). Subsequently, a nasal spray was administered which was either introduced as a means to reduce the negative emotional reactions to the angry facial expressions (OLP group) or to improve the electrophysiological recordings (CG). Both groups were informed that the nasal spray did not contain any ingredients other than saline and water.

The analysis of the EEG data focused on the LPP, which is evident as a broad superior-posterior positivity during the presentation of affective images. The LPP interval starts around 400 ms after picture onset and may extend to the end of picture presentation (e.g. 6000 ms)^13^. Emotionally engaging stimuli elicit larger LPPs compared to neutral (non-arousing) stimuli. This modulation is maximal over centroparietal areas (for a review see^14^). The LPP (particularly the early LPP component < 1000 ms) has been interpreted as an index of attention allocation to incoming information^15^. It reflects heightened processing for motivationally relevant stimuli^16^.

The increase in attention toward, and processing of, intrinsically motivating stimuli has been linked to memory encoding and storage. Higher LPP amplitudes are correlated with better memory for pictures^17^. In the present investigation, an unannounced memory task was conducted after the picture viewing. Recognition performance for the displayed faces was compared between the OLP group and the control group.

LPP modulation has also been found to accompany emotion regulation processes (for a summary see^15^). The use of attentional and emotion regulation strategies (e.g., changes of attentional focus, cognitive reappraisal) reduces the magnitude of the LPP. These changes can be seen in frontal brain areas^18^ as well as in centroparietal areas^19^. In the current study, we analyzed late positivity in a centroparietal and frontal cluster and investigated early (400–1000 ms) and late LPPs (1000–6000 ms). The preregistered hypotheses were:

- The OLP reduces LPP amplitudes, particularly for anger expressions.
- The participants of the OLP group rate the anger expressions as less intense and feel less negative and aroused than the CG.

As an exploratory research question, it was investigated whether OLP treatment would reduce the number of recognized (anger) images. Moreover, we investigated possible associations between the effectiveness ratings for the placebo, affective ratings, and LPP amplitudes.

## Results

### Ratings

#### Affective facial expressions

**Figure 1:**
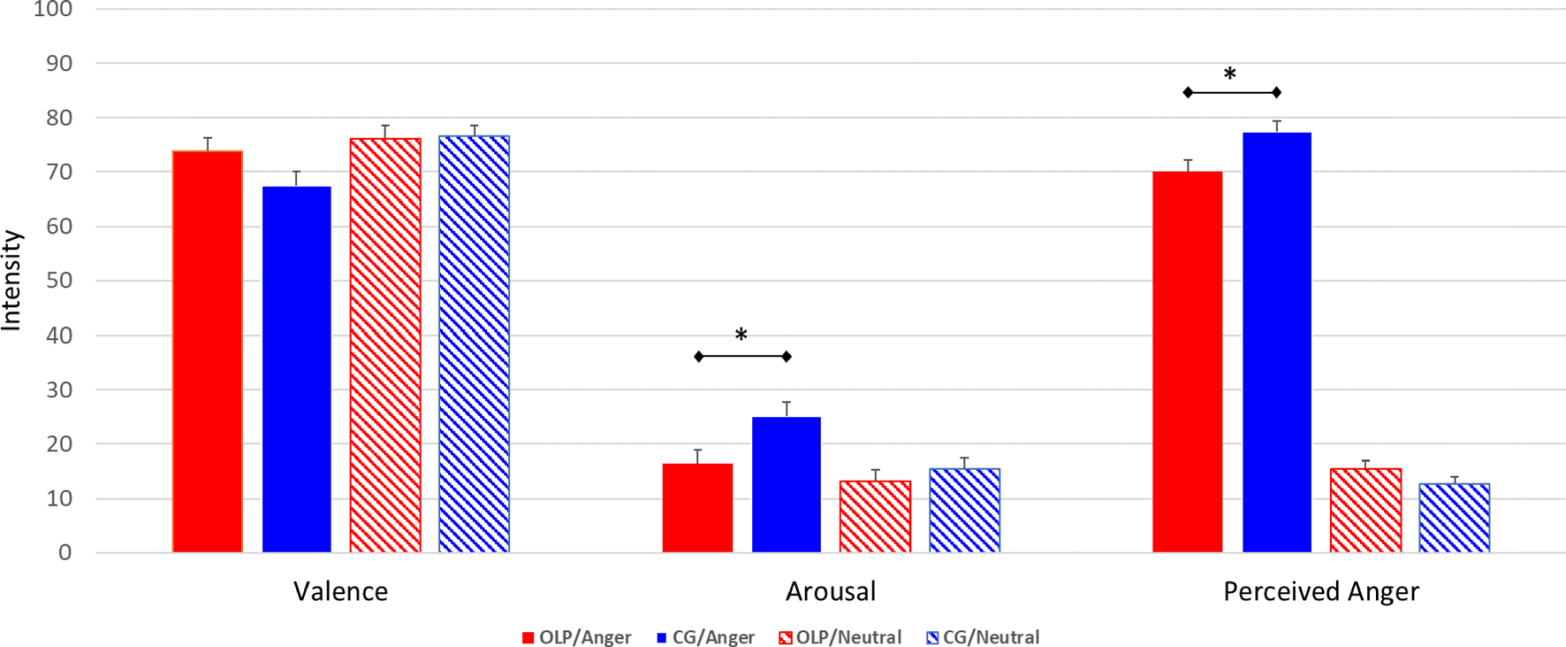
Means and standard errors for picture ratings (valence, arousal, anger intensity) by the open-label placebo (OLP) group and the control group (CG).

The analysis of variance (ANOVA) revealed a significant main effect for Face (angry, neutral) and a significant interaction Group (OLP, CG) × Face (angry, neutral) on ratings for valence, arousal, and perceived anger intensity (Table 1). The participants in the OLP group (compared to the CG) reported feeling less aroused and perceived less anger expressed in angry faces (arousal *t*(101) = − 2.41, *p* = 0.018, d = 0.48; anger: *t*(101) =  − 2.53, *p* = 0.013; d = 0.50). The post-hoc comparison for valence did not reach statistical significance (*t*(101) = 1.73, *p* = 0.086; d = 0.34). The affective ratings for the neutral faces did not differ between the groups (all p > 0.18; Fig. 1).

**Table 1:** Results of the mixed-factorial analyses of variance for self-reports of valence, arousal, and perceived anger intensity by the open-label placebo (OLP) group and the control group (CG).

Effect	F-statistic
**Valence**	
Main effect group (OLP, CG)	F(1,101) = 0.83, p = .36, d = .18
Main effect face (angry, neutral)	F(1,101) = 25.85, p < .001, d = 10
Interaction group × face	F(1,101) = 8.99, p = .003, d = .58
**Arousal**	
Main effect group (OLP, CG)	F(1,101) = 3.12, p = .08, d = .36
Main effect face (angry, neutral)	F(1,101) = 31.78, p < .001, d = 1.12
Interaction group × face	F(1,101) = 8.00, p = .006, d = .54
**Anger intensity**	
Main effect group (OLP, CG)	F(1,101) = 1.69, p = .20, d = .28
Main effect face (angry, neutral)	F(1,101) = 1180.09, p < .001, d = 6.78
Interaction group × face	F(1,101) = 8.34, p = .005, d = .58

#### Presentations

The two groups rated the presentations (about placebos/affective neuroscience) as very interesting (OLP group: M = 8.25, SD = 1.05, CG: M = 8.18, SD = 1.68; p = 0.79, d = 0.06).

#### Emotional state before the picture viewing

Both groups rated their emotional state (valence) before the viewing of the facial expressions as very positive (OLP: M = 8.50, SD = 1.29, CG: M = 8.73, SD = 1.23, p = 0.23, d = 0.18).

#### Effectiveness ratings for the nasal spray

Before the experiment, the OLP group rated the expected effectiveness of the nasal spray (0 = not effective; 10 = very effective) with M = 6.08 (SD = 2.10). After the experiment the perceived effectiveness was rated lower (M = 4.40, SD = 2.46, t(51) = 4.95, p < 0.001, d = 3.06). The CG gave a rating of assumed effectiveness for the nasal spray (after the experiment) of M = 4.47 (SD = 2.63).

### Late positive potentials (LPPs)

**Figure 2:**
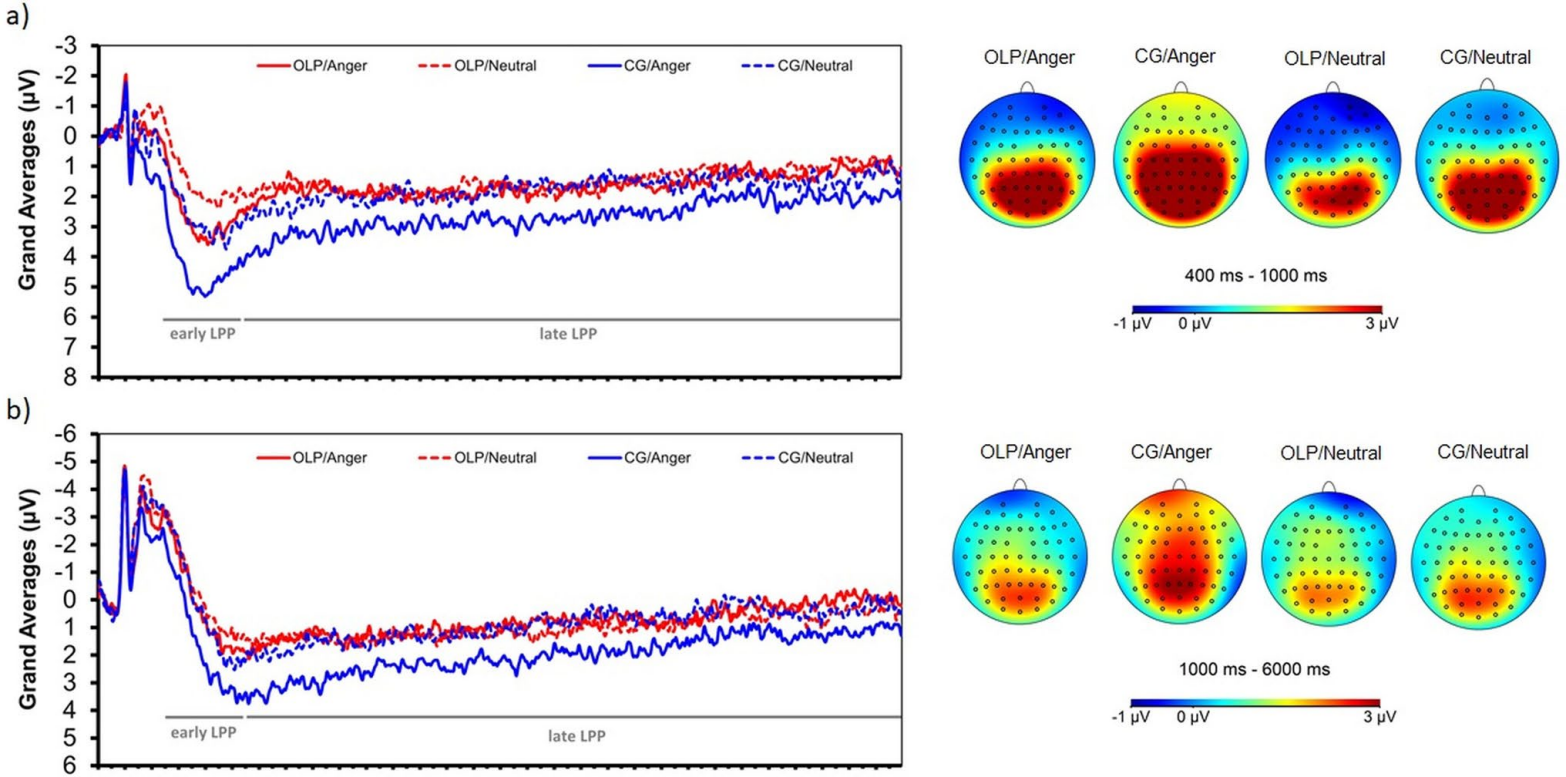
Grand averages (and headmaps) for angry and neutral faces in the open-label placebo group (OLP) and the control group (CG) across the centroparietal cluster (**a**) and the frontal cluster (**b**).

The descriptive statistics (means, standard errors) for the LPP amplitudes (per group and picture category) are depicted in Table 2. Grand averages and headmaps are dispalyed in Fig. 2.

**Table 2:** Means and standard errors for late positive potentials (early LPPs: 400–1000 ms) and late LPPs (1000–6000 ms) in the open-label placebo (OLP) group and the control group (CG).

	OLP	CG
	M (SEM)	M (SEM)
**Angry faces**		
Early LPP frontal	0.27 (0.48)	1.65 (0.39)
Late LPP frontal	0.76 (0.33)	1.95 (0.26)
Early LPP centroparietal	2.74 (0.42)	4.49 (0.45)
Late LPP centroparietal	1.60 (0.33)	2.62 (0.26)
**Neutral faces**		
Early LPP frontal	− 0.18 (0.41)	0.35 (0.37)
Late LPP frontal	0.97 (0.28)	0.87 (0.27)
Early LPP centroparietal	1.63 (0.36)	2.75 (0.41)
Late LPP centroparietal	1.47 (0.26)	1.72 (0.27)

#### Centroparietal cluster

*Early LPP* (400–1000 ms)*:* The effects for Group (F(1,101) = 7.11, p = 0.009, d = 0.54) and Face (F(1,101) = 41.12, p < 0.001, d = 1.28) were statistically significant. Angry facial expressions were associated with greater LPP amplitudes (Table 2). The OLP group displayed lower amplitudes than the CG. The interaction Group x Face was not significant (F(1,101) = 1.96, p = 0.16, d = 0.28).

*Late LPP* (1000–6000 ms)*:* The main effect for Face was statistically significant (F(1,101) = 5.46 , p = 0.02, d = 0.46). Angry facial expressions were associated with greater LPP amplitudes (Table 2). The Group effect (F(1,101) = 3.61, p = 0.06; d = 0.40) and the interaction Group x Face (F(1,101) = 3.02, p = 0.09; d = 0.36) were not statistically significant.

#### Frontal cluster 

*Early LPP* (400–1000 ms)*:* The main effect for Face was statistically significant (F(1,101) = 14.86, p < 0.001, d = 0.78). Angry facial expressions were associated with greater LPP amplitudes (Table 2). The Group effect (F(1,101) = 3.10, p = 0.08; d = 0.36) and the interaction Group x Face (F(1,101) = 3.53, p = 0.06; d = 0.40) were not statistically significant.

*Late LPP* (1000–6000 ms)*:* The main effect for Face (F(1,101) = 3.81, p = 0.040, d = 0.40) and the interaction Group x Face were statistically significant (F(1,101) = 8.50, p = 0.004; d = 0.58). Angry facial expressions were associated with lower LPP amplitudes in the OLP group compared to the CG (t(101) = 2.86, p = 0.005, d = 0.56). The Group effect was not statistically significant (F(1,101) = 2.64, p = 0.11; d = 0.36).

#### Exploratory analyses

##### Memory performance

The ANOVA revealed no statistically significant effects (Face: F(1,101) = 0.81, p = 0.37, d = 0.20; Group: F(1,101) = 0.56, p = 0.46, d = 0.20, Face x Group: F(1,101) = 1.67, p = 0.19, d = 0.28). Both groups showed a very good memory performance (i.e. percentage of correct identifications: angry faces: OLP: M = 85%, SD = 17%; CG: M = 86%, SD = 18%; neutral faces: OLP: M = 86%, SD = 19%; CG: 80%, SD = 20%).

##### Correlation analyses

In the OLP group, the expected effectiveness for the placebo was not associated with early/ late LPP amplitudes (in frontal/ centroparietal clusters) and the picture ratings (valence, arousal, anger intensity). The perceived placebo effectiveness after the experiment was negatively correlated with the ratings for perceived anger intensity in angry faces (r = − 0.30, p = 0.03) and positively with the valence ratings for the neutral faces (r = 0.30, p = 0.03). All other correlations were not statistically significant.

## Discussion

The present study demonstrated that OLP treatment can change the neural processing and evaluation of images depicting facial anger expressions.

Consistent with a previous OLP/EEG study^11^ with similar rationale and instructions, the placebo decreased measures of emotional distress. The OLP group reported less arousal while looking at the angry faces compared to the control group and gave lower intensity estimates for the anger expressions. The ratings for valence were in the predicted direction. The OLP group gave more positive ratings than the CG. The difference was marginally significant. The evaluation of the neutral faces (valence, arousal, perceived anger intensity) did not differ between the two groups. Thus, the OLP specifically altered the subjective anger perception and the affective response (arousal).

In line with a previous ERP study^11^, the OLP reduced the LPP amplitude in a centroparietal cluster to both neutral and negative stimuli. In the present experiment, the early LPP amplitude (400–1000 ms) was lower in the OLP group compared to the control group. In contrast, Guevarra et al.^11^ detected the placebo effect in the late LPP window (1000–6000 ms after picture onset). This difference concerning the timing of the effect may be related to the complexity of the stimulus material. Affective scenes usually have a higher perceptual complexity compared to pictures with facial expressions. Such differences have been associated with the modulation of the early LPP. Bradley et al.^16^ demonstrated that pictures with simple figure-ground compositions elicited larger positivity in an early LPP window (400–700 ms) than complex scenes. However, it should be noted that the OLP effect for the late LPP was in the predicted direction and marginally significant (p = 0.06).

The OLP group of the current study additionally showed reduced amplitudes of the late frontal LPP to angry facial expressions compared to the CG. Thus, the placebo not only exerted a dampening effect on emotional reactivity but specifically changed the processing of the anger cues.

LPP modulation has been associated with the use of emotion regulation strategies (attention shifting, cognitive reappraisal). In an experiment by Krolak-Salmon et al.^20^, the participants were given different tasks during the viewing of facial expressions that involved changing the attentional focus. They were instructed to either attend to the expression (counting surprised faces) or the sex of the faces. Attention to expression increased the LPP. In contrast, the distraction from an affective image leads to reduced magnitudes of the LPP^21^.

Cognitive reappraisal to change the emotional meaning of a stimulus very consistently has elicited LPP modulation. Reappraisal of unpleasant images more neutrally reduces late positivity^19,22^. In a study by Foti and Hajcak^19^, verbal affective descriptions preceding arousing images changed the LPP (400–3000 ms after picture onset). The introduction of the pictures (e.g., images with car accidents) in a negative way (‘Two people died in this horrendous car crash’) was associated with a greater LPP amplitude than a more neutral description (‘No one was seriously injured in this car accident’). This effect occurred across both frontal and centroparietal recoding sites. Moreover, the neutral descriptions reduced the arousal ratings for the subsequently shown unpleasant pictures.

In experiments with functional magnetic resonance imaging (fMRI), the successful implementation of reappraisal during the viewing of unpleasant images has been accompanied by activation of the prefrontal cortex^23,24^. This cortical region is one neural source of the frontal LPP^25^.

Thus, the comparison of the ERP findings from the current OLP study and results from reappraisal experiments^19^ share striking similarities. Reappraisal has been conceptualized as a regulation strategy that influences the emotion-generative process. The participants are provided with instructions to reinterpret emotional stimuli. For example, they try to internally remove themselves from the emotional context presented (‘emotionally detached reappraisal’) or they evaluate the material more positively (‘positive reappraisal’). In a study by Moser et al.^26^, the participants were asked to imagine that the pictured scene improved and to think of the image in a more positive light to decrease the intensity of their negative feelings. Positive reappraisal of distress-inducing images increased the frontocentral LPP (early and late) and decreased the late parietal LPP. Within this context, Ashar et al.^27^ has argued that placebo effects are largely shaped by psychological appraisals, which refer to constructed interpretations of the meaning of events in a given context. Placebos can change appraisals of incoming distressing stimuli.

In their review article, Colloca and Horwick^9^ mention further mechanisms that are connected with the OLP response: pharmacological memory, partial reinforcement, and classical conditioning. These mechanisms, however, cannot explain the effects of the present study since the participants had no prior experience with the inert substance (the nasal spray). A relatively new approach attempts to explain OLP effects with ‘embodied cognition’^28–30^. This concept implies that OLPs stimulate the body to react in a way that subsequently leads to specific cognitions. Thus, cognitions are shaped by aspects of the body including motor actions, such as the intake of the placebo. Models for embodied cognition emphasize that cognition has its roots in motor behavior. This aspect could distinguish ‘pure’ cognitive reappraisal from embodied cognition effects induced by OLPs. Future research is needed to further elucidate the underlying mechanism of OLPs. This can be achieved by directly comparing different strategies for emotion regulation (e.g., OLP vs. reappraisal) and OLP approaches that include more or less dominant motor components (e.g., taking 10 vs. only one placebo pill).

Further, attentional processes associated with OLP treatment require further investigation. It has been shown that changes in spatial attention can function to decrease the emotional response while viewing affective pictures. Van Reekum et al.^31^ measured gaze fixation and found that when participants were instructed to decrease emotions to an unpleasant image, they frequently looked at non-emotional areas of the picture. Therefore, future OLP research on affective picture processing should include eye-tracking methods. Finally, fMRI could provide important information on localized activity and functional connectivity during OLP treatment.

Several critical aspects and limitations of the present study need to be mentioned. We studied a sample of female university students. Therefore, the findings cannot be generalized to other groups. We detected short-term effects of OLP treatment on event-related potentials and self-report measures. The placebo did not influence memory performance as reflected by the number of correctly recognized facial expressions (hits). On the one hand, this finding could indicate that the placebo-induced changes in emotional processing were not sufficient to affect the retrieval of the stimuli. On the other hand, the memory task was easy with a mean percentage of 84% hits, which makes the detection of small placebo effects more difficult. No significant OLP effects on cognitive performance (but reductions in emotional distress) have been reported before^12^.

We investigated a group that was optimistic concerning OLP effects. The expected effectiveness for the placebo was above-average before the experiment. After the experiment, the ratings for the perceived effectiveness decreased. This response could be interpreted as a disappointment. In future investigations, treatment expectancy and rationale credibility should be examined more closely (e.g., How logical does the intervention offered to you seem?). Ratings of expected effectiveness for the OLP were not correlated with neural measures and self-reports. In their review article, Kaptchuk and Miller^29^ questioned that expectation can adequately explain the benefits seen in OLP trials. However, greater perceived effectiveness for the placebo was associated with lower intensity ratings for displayed anger in the angry faces.

We also like to summarize the assets of the present investigation, which included blinded experimenters and accessors as well as a control group that was not aware of the placebo arm of the study. Moreover, interactions of the experimenters and the two groups (OLP, CG) were structurally similar, the instructions were standardized (partly video-recorded) and the presented information was rated as equally interesting by both groups.

## Methods

### Participants

A total of 109 right-handed females were recruited from a nonclinical sample at a university and through social media. The participants were university students (77%) or white-collar workers (13%). Inclusion criteria for participation included female sex and age ≥ 18 years. Exclusion criteria were self-reported diagnoses of mental illnesses, neurological disorders, and psychotropic medication. Data from six participants were removed from the analysis due to excessive EEG artifacts (i.e. less than 70% artifact-free trials). The final sample included 103 females (M_age_ = 22.48 years, SD = 3.35; all Caucasian, right-handed). The participants were randomly assigned to the OLP group (M_age_ = 22.46 years, SD = 3.01) or the CG (M_age_ = 22.49 years, SD = 3.70). The sample was restricted to females because of gender effects in the context of emotional processing and placebo reactivity^32^.

The experiment complied with all relevant ethical guidelines and regulations involving human participants and was approved by the ethics committee of the University of Graz (Austria; GZ. 39/98/63 ex 2020/21). All participants provided informed consent before participating. Eligible participants were scheduled to come into the lab.

### Experimental design and procedure

The study was preregistered on the Open Science Framework (https://osf.io/cxfgb/?view_only=5f4c6d564) on April 30th, 2021, and conducted between May, 25th and August, 30th 2021 at the University of Graz (Austria). Additionally, the study was registered as a clinical trial on the German Clinical Trials Register (DRKS00028129; February, 18th, 2022).

The participants were invited to an EEG study on affective processing (no information about placebos was provided in the invitation). In the preparation room, a female investigator used a random number table to assign the participants to the OLP group or the control group. Both groups viewed a presentation (15 PowerPoint slides with figures and text; no audio; fixed timing per slide: 30 s; see supplementary material). Those in the OLP group received information about the neurobiological effects of placebos with a focus on affective processing (findings from EEG and fMRI studies). The control group looked at a presentation about affective neuroscience (findings from EEG and fMRI studies). Both presentations were comparable in the number of slides, figures, number of words). Afterwards, the participants rated the presentation (0 = not interesting; 10 = very interesting) and their current affective state (0 = not positive; 10 = very positive).

Subsequently, a brief video (duration: two minutes; female presenter) introduced the nasal spray. It was clearly stated that the nasal spray did not contain any ingredients other than saline and water. The OLP group was informed that the nasal spray could help reduce the emotional reactions while viewing images with facial expressions of anger, while the CG was told that the spray would improve the EEG recording. The information was summarized by the experimenter, who helped to deliver the nasal spray once to each nostril. The participants of the OLP group evaluated their expectations concerning the effectiveness of OLPs (‘What do you think? How effective will the OLP be?’ 0 = not effective; 10 very effective).

Then, the participants were brought to the EEG lab. Two female experimenters, who were not informed about the group assignment, conducted the EEG experiment. At the end of the EEG experiment, participants were asked to rate the effectiveness of the nasal spray (‘How effective do you think was the nasal spray?’ 0 = not effective; 10 very effective).

### Image viewing task

The participants viewed a total of 60 images from the Karolinska Directed Faces^33^. Thirty images depicted angry facial expressions; 30 images neutral facial expressions; 50% of the faces were male; 50% female). The pictures were presented in a randomized order. In each trial, participants first viewed a blank screen (500 ms), a fixation cross (500 ms), and then a picture with a facial expression (6000 ms). Eight pictures (four angry/four neutral expressions; 50% male/female) were rated according to valence, arousal, and perceived intensity of anger (0–100; 0 = I feel not pleasant, calm, I perceive no anger; 100: I feel very pleasant, aroused, I perceive intense anger). The pictures for the ratings had been randomly selected before the experiment; the ratings had to be provided at random time points during the experiment.

### Electrophysiological recording and data analyses

Continuous EEG activity was recorded using the actiCHamp system (actiCHamp, Brain Products GmbH, Gilching, Germany) with 63 active actiCAP snap electrodes (according to the 10–10 system) and the BrainVision Recorder (version 1.21). The reference electrode was placed on position FCz, the ground electrode on position FPz. An electrolyte gel was applied to each electrode to keep electrode impedances below 10 kΩ. The EEG was recorded with a sampling rate of 2500 Hz and a passband of 0.016–1000 Hz. For raw data analysis, the BrainVision Analyzer (version 2.2.1) was used. The sampling rate was changed to 250 Hz. The data were re-referenced to linked mastoid electrodes (i.e., TP9, TP10). Artifacts due to eye movements were corrected via the implemented ICA ocular correction software—only components corresponding to horizontal and vertical eye movements were selected based on the correspondence of their shape, timing, and topography. Further artifact episodes were excluded after visual inspection. Six participants were excluded from the analysis due to a large number of artifacts (< 70% of artifact-free segments). For the remaining participants, the percentage of artifact-free trials did not differ between groups (OLP: M = 93.53%, SD = 7.99; CG: M = 93.50%, SD = 7.75 and picture types (anger: 93.51%, SD = 7.40; neutral: M = 93.53%, SD = 6.53) (all p > 0.05).

Data were segmented in 6200 ms intervals (200 ms pre-stimulus onset, 6000 ms post-stimulus onset) and corrected to the 200 ms pre-stimulus baseline. An offline high-pass (0.01 Hz) and low-pass filter (cut-off frequency 30 Hz, roll-off 24 dB/octave) were applied. Data were averaged for all groups and conditions separately. Based on previous literature and visual inspection of grand average waveforms, we extracted ERPs for the time windows 400–1000 ms (early LPP) and 1000–6000 ms (late LPP) after picture onset. Mean amplitudes were aggregated across a centroparietal cluster (C3, C1, Cz, C2, C4, CP3, CP1, CPz, CP2, CP4, P3, P1, Pz, P2, P4) and a frontal cluster (AF3, AFz, AF4, F3, F1, Fz, F2, F4, FC3, FC1, FC2, FC4).

### Memory task

After viewing the pictures, an unannounced memory task was conducted with 16 pictures (8 angry expressions, 8 neutral expressions; 50% males, 50% females). Eight of the pictures had been shown in the experiment; the other pictures were new distractors. The pictures had been randomly selected before the experiment (and were different from the evaluated pictures (affective ratings) during the experiment). The participants were asked to decide whether they had seen the picture in the experiment (yes/no).

### Data analysis

All statistical analyses were performed with SPSS (version 28). The investigator who analyzed the data collected from the study was not aware of the treatment applied to the groups. Mixed-factorial analyses of variance (ANOVAs) were computed with Group (OLP, CG) as a between-subjects factor, and Face (angry, neutral) as a within-subjects factor for picture ratings, memory performance, and EEG data. Cohen’s d is reported as effect size measure. Significant interaction effects (Group x Face) were followed by t-tests adjusted for multiple comparisons (Bonferroni-Holm).

T-tests compared the groups concerning their ratings for the PowerPoint presentations (rated interest), the emotional state before the experiment, and the perceived effectiveness of the nasal spray. All tests were two-tailed and used a significance level of p < 0.05.

## Supplementary Information

Supplementary Information.
