# Supplementary material for: Changes in neural processing and evaluation of negative facial expressions after administration of an open-label placebo
Source: Sci Rep. 2022 Apr 21;12:6577. doi: 10.1038/s41598-022-10567-4 (PMC9023441; doi:10.1038/s41598-022-10567-4)
Supplement: Supplementary file 1 — Supplementary Information. [file 41598_2022_10567_MOESM1_ESM.docx]

**Supplementary material: Instructions and presentations created and translated by the authors**

**1. Translated transcripts of the Power-Point presentations for the Control group and the Open label Placebo group**

**1.1 Open-label placebo (OLP) group: slides**

**Slide 1: Dear participant!**

Thank you for agreeing to participate in this study about the electrocortical processing of affective facial expressions. After the conclusion of this presentation you will be presented with images of men and women. Please view these pictures in the same manner as you would if you were watching TV.

**Slide 2: Placebos**

This study examines the effects of a placebo. Placebos are substances (e.g. pills) or interventions (e.g. sham treatments) that have no direct effect on the symptom being treated.

Nevertheless, placebos can have very positive effects, such as pain relief.

When a pill is administered that looks like a painkiller many people experience pain reduction. Additionally, there are placebo-related physical changes. For example, the body releases specific substances (endogenous opiates), which have been shown pain-relieving effects. Placebos also provoke changes in brain activity among the regions involved in pain processing.

**Slide 3: Scientific Research**

An article (Wager & Atlas, 2015) in the prestigious scientific journal Nature summarized the effects of placebos as follows:

Placebos alter brain activation in those regions of the brain involved in sensory aspects of pain processing (e.g., intensity, type of pain - e.g., dull, stabbing, etc.) and emotional aspects of pain processing (suffering).

**Slide 4: Brain regions that respond to placebo administration**

Visual Cortex: Processing and integration of visual stimulus characteristics

Insula/ Anterior Cingulum: Analysis of emotional/ motivational stimulus components

Prefrontal Cortex: Meaning of emotional stimuli

Thalamus: Sensory gating

Brain stem: Basal components of stimulus processing

**Slide 5: How do placebos work?**

A placebo can be helpful because of positive expectations (you think it will work) and previous learning experiences.

For example, if you use a nasal spray (inactive substance) to relieve pain and you are convinced that it is a pain reliever, the pain may decrease.

The attitude and the belief that a treatment can help is a central mechanism of the placebo effect.

**Slide 6: Important placebo findings**

Placebos can reduce movement symptoms (e.g., tremor, slowness) in patients with Parkinson’s disease (neurodegenerative disease) (Götz et al., 2017)

Athletes who took placebos labeled as performance-enhancing drugs performed objectively better (e.g., ran faster, lifted heavier weights) (Beedie et al., 2009)

Patients with depression experienced a significant reduction in symptoms through placebo treatment (Kelley et al., 2012)

**Slide 7: Placebo effects on emotional processes**

Placebos can also reduce negative emotions in healthy people.

A study conducted by the Department of Clinical Psychology at the University of Graz showed that a placebo (introduced as herbal anti-nausea medicine) reduced the intensity of disgust experienced when viewing disgusting images (Schienle et al., 2014).

**Slide 8: Additionally, the placebo reduced brain activity**a) in the visual cortex (the pictures were viewed ‚differently‘)
b) in the insula (important region for disgust processing)

**Slide 9: A new approach**

In the placebo studies described, the participants were not truthfully informed about the treatment (they never received a real drug).

At first, doctors and scientists thought it was necessary to deceive people in order for placebos to work. This is not entirely true however.

New research has shown that deception is NOT necessary. A placebo can work even if you know it is a placebo.

Such placebos are also known as OPEN LABEL PLACEBOS.

**Slide 10: Open-Label Placebos: Studies**

In a meta-analysis (summary of findings from individual studies) by Charlesworth et al. (2017) it was shown that placebos WITHOUT deception can reduce symptoms of a wide variety of diseases, such as

- Irritable bowel syndrome
- Attention deficit hyperactivity disorder (ADHD)
- Chronic back pain
- Depression

**Slide 11: A study published in Nature (Guevarra et al., 2020) showed that**

a placebo administered WITHOUT deception (open label placebo: saline nasal spray) reduced emotional distress in participants while viewing unpleasant images.

In addition, indicators of emotional distress were reduced in the electroencephalogram (EEG). The amplitudes of so-called late positive potentials decreased after the administration of the placebo.

**Slide 12: Image of Late Positive Potentials**

**Slide 13: Summary**

You have received information about placebos and placebo effects. The following points are important:

Placebos are inactive substances or procedures that can make people feel better.

Placebos have beneficial effects on a variety of conditions, symptoms, and disorders such as negative emotions, pain, and depression.

Placebos not only change how a person feels subjectively, but also behavior and somatic processes (e.g. brain activation).

Placebos can work even if you know you are taking a placebo. Studies with open-label placebos have produced similar effects as 'traditional' placebos.

**Slide 14: References**

Beedie & Foad (2009) The Placebo Effect in Sports Performance. A Brief Review. Sports Medicine 39(4):313-29.

Charlesworth., J.E.G., Petkovic, G., Kelley, J.M., Hunter, M., Onakpoya, I., Roberts, N., Miller, F.G., Howick, J., 2017. Effects of placebos without deception compared with no treatment: A systematic review and meta-analysis. Journal of Evidence-Based Medicine 10, 97–107.

Goetz, C. G., Laska, E., Hicking, C., Damier, P., Müller, T., Nutt, J., Warren Olanow, C., Rascol, O., & Russ, H. Placebo influences on dyskinesia in Parkinson's disease. Movement disorders: official journal of the Movement Disorder Society, 23(5), 700–707.

Guevarra, D. A., Moser, J. S., Wager, T. D., & Kross, E. (2020). Placebos without deception reduce self-report and neural measures of emotional distress. Nature communications, 11(1), 1-8.

Kelley, J.M., Kaptchuk, T.J., Cusin, C., Lipkin, S., Fava, M., 2012. Open-label placebo for major depressive disorder: a pilot randomized controlled trial. Psychother. Psychosom. 81 (5), 312–314.

Schienle A, Übel S, Schöngaßner F, Ille R, Scharmüller W. Disgust regulation via placebo: an fMRI study. Soc Cogn Affect Neurosci. 2014 Jul;9(7):985-90. doi: 10.1093/scan/nst072.

Wager TD, Atlas LY. The neuroscience of placebo effects: connecting context, learning and health. Nat Rev Neurosci. 2015 Jul;16(7):403-18. doi: 10.1038/nrn3976. PMID: 26087681; PMCID: PMC6013051.

**1.2 Control group: slides**

**Slide 1: Dear participant!**

Thank you for agreeing to participate in this study about the electrocortical processing of affective facial expressions. After the conclusion of this presentation you will be presented with images of men and women. Please view these pictures in the same manner as you would if you were watching TV.

**Slide 2: Affective neuroscience**

Affective neuroscience examines emotional and motivational influences on information processing in different areas. The research field focuses on the underlying neural as well as peripheral physiological mechanisms of these processes.

Examples of research topics include:

• Emotional and motivational influences on sensory and cognitive processes

• Integration of social signals

• Neural correlates of emotional processes in mental disorders

**Slide 3: How are emotional stimuli processed?**

Emotional events are made up of information from different modalities. When, for example, a person is threatened by a dog, this person sees the dog and at the same time hears the growling and barking. The threat level of the situation can be recognized and classified only based on the integration of information.

Numerous studies have shown that affective pictures attract motivated attention which facilitates perceptual processing of these survival-relevant stimuli.

**Slide 4: Processing of emotional stimuli in the brain**

Visual Cortex: Processing and integration of visual stimulus characteristics

Insula/ Anterior Cingulum: Analysis of emotional/ motivational stimulus components

Prefrontal Cortex: Meaning of emotional stimuli

Thalamus: Sensory gating

Brain stem: Basal components of stimulus processing

**Slide 5: Scientific Research**

Research methods in Affective Neuroscience include functional magnetic resonance imaging (fMRI) and electroencephalography (EEG).

FMRI indirectly records which brain areas are activated by measuring blood oxygenation (oxygen concentration). The method has high spatial resolution and provides information about where the activation takes place in the brain.

The EEG has a high temporal resolution (in the range of milliseconds) and records the electrical activity in the brain (cumulative potentials).

**Slide 6: Important Findings**

The processing of emotional stimuli is surprisingly fast. After only a few milliseconds (approx. 200 ms) the brain can distinguish between an emotional stimulus and a neutral stimulus.

Emotional processing can also take place without conscious perception. Affective stimuli are sufficiently analyzed in subcortical regions to motivate functional behaviors (e.g. escape behavior)

It is unlikely that a specific region of the brain or a ‘neural module’ is permanently dedicated to mediate only one affective function. New findings have indicated that a module can exist in multiple neurobiological states with different affective functions (Berridge, Nature Reviews Neuroscience, 2019)

**Slide 7: Influence of personality traits on affective processing**

When we look at emotional images, specific personality traits play a role in influencing how intense the emotions are felt.

A study conducted by the Department of Clinical Psychology at the University of Graz showed that brain activity differed between people with very high vs. low levels of disgust propensity (temporally stable tendency to experience disgust across different situations) (Schienle et al., 2014)

**Slide 8: Individuals with high vs. low disgust propensity differ in their brain activiation in**a) the visual cortex (the pictures are viewed ‚differently‘)
b) the insula (important region for disgust processing)

**Slide 9: A new approach**

There are many new developments in the field of fMRI. Brain activation is not only examined among certain brain regions, but also activation networks can be mapped. It is possible to study so-called functional connectivity, i.e. brain regions that show concurrent activation indicating the exchange of information.

In causal connectivity analyses, the direction of information exchange is examined, i.e. which brain region influences another brain region.

Connectivity analyses are also possible with EEG data

**Slide 10: Networks**

The brain also shows specific activation patterns during resting states: ‚Resting State Connectivity‘. Large-scale studies have shown that these activation patterns can be assigned to specific systems (networks):

The Default Network is involved in self-referential processing and mentalizing (e.g., thoughts you have about yourself)

The Salience Network integrates interoceptive, autonomous und emotional information (e.g., when you have a certain ‚gut feeling‘)

**Slide 11:** **An EEG study (Meule et al., 2013) published in Frontiers showed that**

images depicting food were rated as more positive than non-food images. Particularly high-calorie food (sweets) was rated as more pleasant and more appetizing than low-calorie food (salad).

The differential emotional/ motivation meaning of the stimuli can also be seen in the electroencephalogram (EEG). Amplitudes of the so-called Late Positive Potential were increased for food stimuli.

**Slide 12: Image of Late Positive Potentials**

**Slide 13: Summary**

You have received information about the research field of Affective Neuroscience. The following points are important:

Affective stimuli are preferentially processed in emotion-relevant but also in visual centers of the brain.

EEG and fMRI are two methods to investigate emotional processes with either high temporal or high spatial resolution.

Affective stimuli can be processed subconsciously and personality traits influence the neural processing of these stimuli.

The brain is emotionally dynamic: the same brain region can have different emotional functions in different situations.

**Slide 14: References**

Berridge, K.C. Affective valence in the brain: modules or modes?. Nat Rev Neurosci 20, 225–234 (2019).

Meule, A., Kübler, A., & Blechert, J. (2013). Time course of electrocortical food-cue responses during cognitive regulation of craving. Frontiers in Psychology, 4, 669.

Schienle, A., Übel, S., Schöngaßner, F., Ille, R., & Scharmüller, W. (2014). Disgust regulation: an fMRI study. Social Cognitive and Affective Neuroscience, 9(7), 985-990.

Wager, T. D., & Atlas, L. Y. (2015). Neuroscience: connecting context, learning and health. Nature Reviews Neuroscience, 16(7), 403-418.

**2. Translated transcripts of the video instructions for the control group and the Open-Label Placebo group**

**2.1 Instruction for the Open-label placebo group**

Dear participants,

You have just read that placebos, which consist of pharmacologically inactive substances, can significantly reduce various symptoms. Scientific studies have shown that placebos can reduce stress, dampen aggression and increase trust in others. Negative emotions such as fear or sadness can also be alleviated.

You have also read that placebos can do more than just influence your feelings on a subjective level. Placebos can change your behavior and even your brain activity. Placebo responses can be either based on learning experiences or based on positive expectations and beliefs that the treatment will be effective. Placebos can be effective when we expect to get better after taking them.

New research has shown that placebos work even when people know they are taking a placebo.

You will now receive a placebo in the form of a nasal spray with a saline solution. Afterwards, you will be asked to look at pictures showing male and female portraits. Please view these pictures in the same manner as you would if you were watching TV. The placebo can help to reduce the intensity of the negative facial expressions and your negative feelings.

**2.2 Instruction for the Control group**

Dear participants,

You have just received information about the research field of Affective Neuroscience. Scientific studies have shown that emotional stimuli can be perceived unconsciously and that emotional events are not only processed in emotion-relevant brain structures, but also in visual centers.

Affective processing can be examined particularly effectively using EEG because of its high temporal resolution. It is also possible to measure which brain regions show concurrent activation and how they exchange information with each other. This is referred to as ‘connectivity’.

You will now receive a nasal spray with a saline solution to obtain optimal physiological recordings. Your nasal cavities are covered with a membrane that is regenerated during the day. This process can interfere with recordings of the electrical signal of the EEG due to so-called movement artifacts, which are produced when you wrinkle your nose.

We therefore would like to ask you to use the nasal spray to clean your nasal cavity.

Afterwards, you will be asked to look at pictures showing male and female portraits. Please view these pictures in the same manner as you would if you were watching TV.
